# Development and evaluation of a lymph node invasion risk prediction model in intermediate- and high-risk prostate cancer patients

**DOI:** 10.2340/1651-226X.2025.43970

**Published:** 2025-10-22

**Authors:** Håkon Ramberg, Manuela Zucknick, Francesco Barletta, Petter Davik, Åsmund Nybøen, Lars Magne Eri, Shivanthe Sivanesan, Knut Håkon Hole, Tord Hompland, Stian Ole Prestbakk, Giorgio Gandaglia, Tone Frost Bathen, Alberto Briganti, Viktor Berge, Kristin Austlid Taskén

**Affiliations:** a Department of Tumor Biology, Institute for Cancer Research, Oslo University Hospital, Oslo, Norway; b Oslo Center for Biostatistics and Epidemiology, University of Oslo, Oslo, Norway; c Unit of Urology/Division of Oncology, Gianfranco Soldera Prostate Cancer Laboratory, IRCCS San Raffaele Scientific Institute, Milan, Italy; d Vita-Salute San Raffaele University, Milan, Italy; e Department of Urology, St Olavs Hospital, Trondheim, Norway; f Department of Clinical and Molecular Medicine (IKOM), Norwegian University of Science and Technology (NTNU), Trondheim, Norway; g Department of Pathology, Oslo University Hospital, Oslo, Norway; h Department of Urology, Oslo University Hospital, Oslo, Norway; i Institute of Clinical Medicine, University of Oslo, Oslo, Norway; j Division of Radiology and Nuclear Medicine, Oslo University Hospital, Oslo, Norway; k Department of Radiation Biology, Institute for Cancer Research, Oslo University Hospital, Oslo, Norway; l Faculty of Medicine, University of Oslo, Oslo, Norway; m Department of Radiology and Nuclear Medicine, St. Olavs Hospital, Trondheim, Norway

**Keywords:** pelvic lymph node dissection, prostate cancer, prediction model, Bayesian logistic regression, lymph node invasion

## Abstract

**Background and purpose:**

Many prostate cancer patients undergoing pelvic lymph node dissection (PLND) have no sign of lymph node invasion (LNI) during final pathological assessment. To improve preoperative staging accuracy, we developed the Oslo model, which estimates the risk of LNI based on clinical, histopathological, and magnetic resonance imaging (MRI) variables.

**Patients/materials and methods:**

We utilized data from 903 prostate cancer patients treated at Oslo University Hospital (OUS) to develop the model using Bayesian logistic regression. The Oslo model was validated with data from 189 patients at IRCCS Ospedale San Raffaele (HRS), 157 from St. Olav’s Hospital, and 231 from OUS. We assessed its performance against the Memorial Sloan Kettering Cancer Centre (MSKCC) and Briganti 2019 nomograms using metrics like AUC, *R*², decision curve analysis, and calibration plots.

**Results:**

The Oslo model outperformed Briganti 2019, demonstrating a higher net benefit and a 10% reduction in interventions at a 7% cutoff. Key variables included clinical T stage on MRI, Prostate Specific Antigen (PSA), prostate volume, International Society of Urological Pathology grade group, and maximum lesion length on MRI. Validation showed strong reliability in the OUS and HRS cohorts but weaker performance in the St. Olav’s cohort. The AUCs were 77% for the Oslo model, 74% for Briganti 2019, and 66% for MSKCC. Limitations include small and heterogeneous validation cohorts.

**Interpretation:**

The Oslo model enhances predictive performance in intermediate- and high-risk patients using easily accessible clinical and MRI data, potentially reducing unnecessary PLND interventions and assisting clinicians in treatment decision-making.

## Introduction

Pathological assessments reveal that 70–90% of patients who undergo extended pelvic lymph node dissection (ePLND) for prostate cancer have negative lymph node status [1]. ePLND increases a patient’s risk of postoperative complications, procedure time, and length of hospital stays [2]. This holds true even in the PSMA-PET era (prostate specific membrane antigen positron emission tomography), where this advanced imaging modality is not always accessible and, more importantly, is characterized by suboptimal sensitivity for nodal invasion in the case of micrometastatic disease and in patients at high risk of lymph node invasion (LNI) [3].

Many predictive models for LNI risk have been developed for patients undergoing robot-assisted laparoscopic prostatectomy (RALP) in order to reduce the number of unnecessary ePLND procedures. Clinical variables collected during diagnosis and preoperative workup such as preoperative PSA, International Society of Urological Pathology biopsy grade group (ISUP GG), and clinical T-stage, are commonly used. Few models include variables from magnetic resonance imaging (MRI) [4, 5].

The main objective of this study was to develop and validate a new prediction model using multivariate Bayesian logistic regression that also included MRI variables, with a focus on the usability of the model. We compared our model with the most used prediction models, MSKCC and Briganti 2019.

## Patients/material and methods

The checklist (Supplementary Methods) from the TRIPOD+AI (Transparent Reporting of a multivariable prediction model for Individual Prognosis Or Diagnosis Artificial Intelligence) statement was used as a guideline in development and validation of the presented prediction models [6].

### Study design and development cohort

The Research Registry of Prostate Cancer at the Oslo University Hospital (OUS), was used to retrospectively identify patients who had undergone a RALP with concomitant ePLND between January 2015 and December 2022. A total of 980 patients underwent RALP and ePLND in this period, with a pN1 prevalence of 27%.

At OUS the European Association of Urology (EAU) guidelines were adhered to in determining eligibility for ePLND from 2015 to 2022. Approximately 80% of all high-risk patients underwent an ePLND [7] and intermediate risk (IR) patients with a predicted risk above 5 or 7% when using the Briganti 2012 or 2019 lymph node prediction nomograms, respectively.

We extracted clinical data, histopathological results, and radiological results (PSA, biopsy ISUP GG, number of biopsy cores, cT-stage (Digital Rectal Examination [DRE]), Age, Body Mass Index (BMI), Prostate Imaging Reporting and Data System (PI-RADS) score, MRI maximum index lesion diameter, MRI T-stage and prostate volume (TRUS or MRI) from the registry. Patients were excluded if they had received neoadjuvant hormonal treatment (*n* = 39), salvage RALP (*n* = 18), or if no MRI was done (*n* = 20) (Supplementary Figure S1).

The whole cohort included 53 patients (5.9%) that had a pre-operative PSMA-PET (9 out of 34 miN0 were pN1 and 5 out 19 miN1 were pN0). Additionally, 81 patients out of 903 were classified with suspicious node (cN1) based on MRI, and of these 28 were pN0. Among the 681 patients classified as cN0, 164 had a positive lymph node metastasis (pN1).

The study was approved by the Regional Ethics Committee of Norway (REK) (REK 563042) and the The Research Registry of Prostate Cancer OUS has been approved by the data protection officer at OUS (PVO 18/07786) and REK (REK 28144).

### External and temporal validation cohorts

To validate our model, two external cohorts of patients who underwent RALP and ePLND at I.R.C.C.S Ospedale San Raffaele (HSR) in Milan Italy and St. Olav’s University Hospital, Trondheim, Norway between 2014 and 2024 were used [4]. The HSR cohort consisted of 189 patients with a pN1 prevalence of 16%. The St. Olav’s cohort consisted of 157 patients with a pN1 prevalence of 25% (REK 2017/576).

A temporal cohort from OUS of patients operated between January 2023 and December 2024, as well as 48 patients from the FuncProst study [8] were collected and used as internal validation. Inclusion criteria were the same as for the cohort used for model development and the cohort consisted of a total of 231 patients with a pN1 prevalence of 28%.

### Biparametric and multiparametric MRI

Prebiopsy MRI became the standard of care for all patients with suspected prostate cancer in Norway in 2015. The MRIs were performed before biopsy at multiple hospitals in the South-Eastern health regions in Norway. Most of the patients had biparametric MRI with T2-weigthed (T2W) and diffusion weighted imaging (DWI) sequences. The guidelines for interpretation, reporting, and staging, outlined in PI-RADS version 2.0 [9], and version 2.1 after 2019, were followed, using the two dominant sequences: T2W and DWI.

### Pelvic lymph node dissection and histopathology examination

All RALP and ePLND procedures in the development cohort were performed at the Department of Urology at OUS. From 2015 to 2022, a bilateral ePLND that encompassed the obturator fossa and external and internal iliac landing areas, was the main procedure for lymph node staging at OUS. All pathological assessments in this study were performed by uropathologists at the Department of Pathology at OUS according to prevailing guidelines [10, 11].

### Sample size calculations

A minimum sample size calculation was done, setting the area under the curve (AUC) of the Receiver Operating Characteristics (ROC) Curve to 0.8 and the prevalence to 0.26 as the baseline to estimate the anticipated *R*^2^ CoxSnell. This was done to assess if our cohort had enough patients and events to meet the criteria set forth by Riley and colleagues [12]. The AUC was chosen based on results from the Briganti 2019 model and the prevalence in the OUS cohort was used. The analysis was done with *R* package pmsampsize version 1.1.2 [13]

### Model development and handling of missing data

An initial Bayesian logistic regression model was developed using all nine predictors available in the OUS cohort (PSA, ISUP grade, clinical *T*-stage, % positive biopsy core, PI-RADS score, MRI *T*-stage, max index lesion length MRI, BMI, and Prostate Volume). Missing values in the dataset were handled by using multiple imputations. The aregImpt function form the rmsb package (version 1.0) in *R* [14] was used with bootstrap and predictive mean matching (PMM) with chained equations to generate 10 imputed datasets. Variable selection was used to reduce the number of variables in the final prediction model. Backward elimination with and without cross-validation using the projpred (version 2.7.0) *R* package [15] was applied in the predictor selection process. The final model development included the use of restricted cubic splines on continuous variables and an interaction term for PSA and prostate volume variables. Models were compared with respect to their estimated expected log-predictive density (ELPD). The leave-one-out cross-validation approach was used as the internal validation method using the rmsb *R* package.

### Validation and recalibration methods

The prediction models chosen for validation were selected according to the guideline recommendations from EAU and American Urological Association (AUA) [16, 17]: MSKCC (without percentage positive biopsy cores, last updated 15th of December 2023) [18] and Briganti 2019 [4]. Both prediction models evaluated included the predictors preoperative PSA, clinical *T*-stage (DRE or MRI), and Gleason score. The Briganti 2019 nomogram also includes length of index lesion from MRI and percentage of positive systematic biopsy cores. The model coefficients were collected from published articles or web resources, and used to calculate the predictive probabilities [4, 18–20]. Area under the curve (AUC) of the Receiver Operating Characteristics (ROC) Curve, *R*^2^, and Brier score were used to assess discrimination and overall fit of the models tested, respectively. The over- and underestimation were tested by using calibration plots to determine the agreement between predicted and observed lymph node status on the OUS cohort. The Briganti 2019 model was further updated by re-calibration or model refitting to the OUS cohort as suggested by Vergouwe and colleagues, using the *R* tutorial by Darren Dahly [21–23]. Decision curve analysis was used to compare net benefits of the different models. Further information about statistical methods and *R* scripts can be found in supplementary appendix B and at hakonrlab.github.io. All statistical analyses were performed using *R* Statistical Software (4.2.2) and RStudio (2022.7.2.576), and its many packages provided by the *R* community [24, 25].

## Results

### Baseline characteristics

The development cohort consisted of 903 patients of which 240 were lymph node positive (pN1, 27%). The clinical characteristics of the cohort are presented in Table 1. The median number of lymph nodes examined in pN0 patients were 16 and 18 for pN1 patients. pN1 patients had a median of two positive nodes. Only 1% of the patients were classified as low risk according to the EAU risk group stratification, whereas 21% were IR and 78% high risk (HR) (Supplementary Table S1). Based on the sample size calculations the cohort should have a minimum of 745 patients with 194 events to precisely estimate an AUC of 0.8 and *R*^2^ Cox-Snell of 0.21 (Supplementary Table S2). Only patients with complete clinical information (pN0 = 582, pN1 = 203) were used in the evaluation of the external models. The distribution of missing data for the variables is shown in Supplementary Figure S2. The baseline characteristics between complete cases and missing cases are shown in Supplementary Table S3.

**Table 1:** Baseline clinical characteristics of the OUS development cohort 2015–2022.

Variable	Lymph node status		*P*
	pN0 (*n* = 663)	pN1 (*n* = 240)	
Age at surgery	68 (63, 72)^1^	67 (63, 72)^1^	0.8^2^
Pre-surgery PSA (ng/mL)	8.80 (6.50, 14.00)	11.70 (7.50, 17.00)	< 0.001^2^
ISUP grade group			< 0.001^3^
≤ 2	52/663 (8%)	16/240 (7%)	
3	180/663 (27%)	54/240 (22%)	
4	263/663 (40%)	64/240 (27%)	
5	168/663 (25%)	106/240 (44%)	
cT-stage (DRE)			< 0.001^3^
T1	300/651 (46%)	69/231 (30%)	
T2	263/651 (40%)	104/231 (45%)	
T3	88/651 (14%)	58/231 (25%)	
Missing	12	9	
No. of biopsy cores	10 (7, 11)	10 (7, 11)	0.6^2^
(Missing)	6	2	
No. of positive biopsy cores	5 (3, 6)	6 (4, 8)	< 0.001^2^
Missing	14	4	
PI-RADS score (MRI)			< 0.001^3^
≤ 3	62/662 (9%)	11/240 (5%)	
4	206/662 (31%)	25/240 (10%)	
5	394/662 (60%)	204/240 (85%)	
Missing	1	0	
cT-stage (MRI)			< 0.001^3^
≤ T2	357/663 (54%)	51/240 (21%)	
T3a	247/663 (37%)	103/240 (43%)	
≥ T3b	59/663 (9%)	86/240 (36%)	
Max lesion length (MRI) (mm)	17 (12, 24)	24 (18, 30)	< 0.001^2^
Missing	59	32	
BMI	26.6 (24, 29)	26.5 (24, 29)	≥ 0.9^2^
Missing	3	1	
Prostate volume (cc)	37 (29, 50)	38 (30, 49)	0.6^2^
Missing	7	0	
No. of removed lymph nodes	16 (12, 20)	18 (13, 23)	< 0.001^2^
No. of positive lymph nodes	0 (0, 0)	2 (1, 3)	< 0.001^2^

OUS: Oslo University Hospital; ISUP: International Society of Urological Pathology; DRE: Digital Rectal Examination; PI-RADS: Prostate Imaging Reporting and Data System; MRI: magnetic resonance imaging; BMI: Body Mass Index.

1 Median (IQR); *n*/*N* (%);

2 Wilcoxon rank sum test;

3 Pearson’s Chi-squared test.

### Development and internal validation of Bayesian logistic prediction models

**Figure 1:**
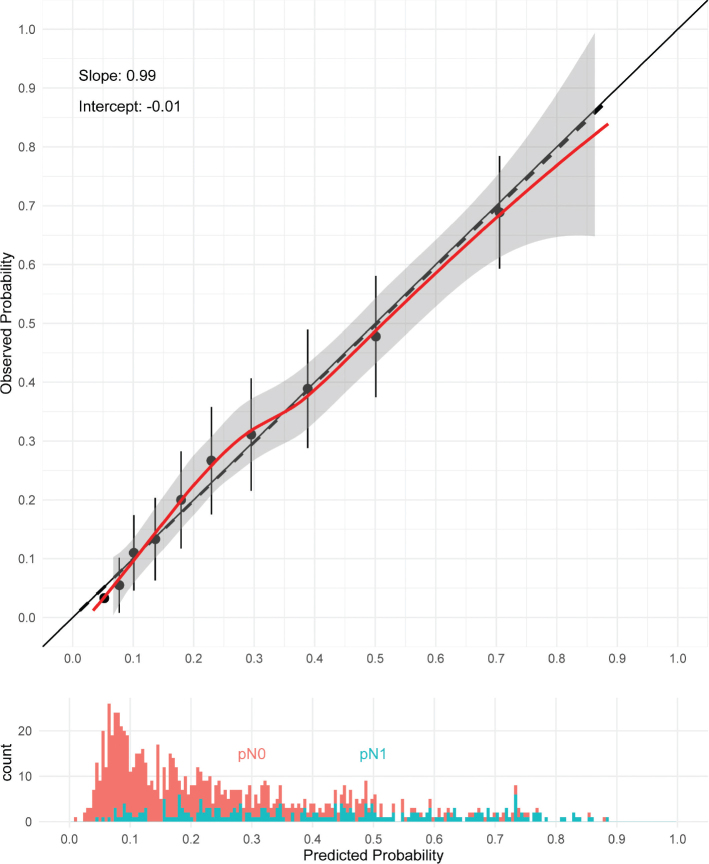
Calibration plot showing the predictive accuracy of the Oslo model using the complete cases from the OUS cohort. The dashed line represents the linear approximation, and the red line is the flexible calibration curve based on locally estimated scatterplot smoothing. The grey shaded area represents the 95% confidence interval. The diagonal black line represents an ideal calibration curve. Histogram shows the distribution of predicted probabilities of pN0 and pN1 patients. OUS: Oslo University Hospital.

The rationale for developing a new LNI prediction model was to use only easily accessible variables and improve performance. Initially, we developed a full model with nine of the relevant predictors available from our cohort (Supplementary Figure S3 and S4). Then by using different predictor selection methods as mentioned previously (Supplementary Figure S5 and S6), and discussions with urologists that use the models in their daily practice, we developed a final model using a pragmatic approach. The prediction variables included in the final Oslo model were PSA, prostate volume, *T*-stage (MRI), maximum lesion length of index tumor (MRI), and highest ISUP GG from biopsies. Table 2 shows the overall performance of both the full and the final Oslo models. Additional information on the coefficient estimates of the final Oslo model is presented in Supplementary Figure S7–S8 and Supplementary Table S4. The final model can be downloaded at hakonrlab.github.io. To compare the two models, we used the ELPD to test the overall compatibility between the models (Supplementary Table S5). The calibration plot of the Oslo model was based on the cohort of patients with complete data (Figure 1).

**Table 2:** Model performance evaluation of Bayesian logistic regression models (95% CI) using the OUS development cohort.

Measure	Full Oslo model	Final Oslo model
AUC	0.78 (0.77, 0.79)	0.77 (0.76, 0.78)
Brier score	0.159 (0.156, 0.163)	0.160 (0.157, 0.163)
Brier score scaled	0.19 (0.17, 0.20)	0.18 (0.17, 0.20)
Explained variation (R^2^)	0.26 (0.22, 0.31)	0.23 (0.18, 0.28)

Full model: PSA, Prostate Volume, ISUP grade, lesion length MRI, T-stage MRI, cT-DRE, BMI, PI-RADS, Percent positive biopsy cores.

Final model: PSA, Prostate Volume, ISUP grade, lesion length MRI, T-stage MRI. OUS: Oslo University Hospital; ISUP: International Society of Urological Pathology; DRE: Digital Rectal Examination; PI-RADS: Prostate Imaging Reporting and Data System; MRI: magnetic resonance imaging; BMI: Body Mass Index.

### External and temporal validation of the Oslo model

**Figure 2:**
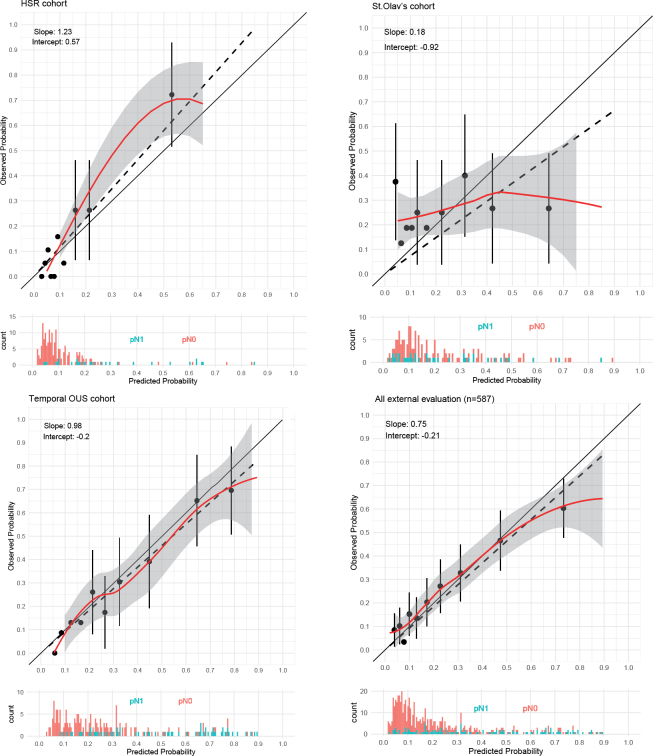
Calibration plots of the external and temporal validation cohorts. The dashed line represents the linear approximation, and the red line is the flexible calibration curve based on locally estimated scatterplot smoothing. The grey shaded area represents the 95% confidence interval. The diagonal black line represents an ideal calibration curve. Histogram shows the distribution of predicted probabilities of pN0 and pN1 patients.

To further validate our model, three external cohorts: two geographical validation cohorts from HSR and St. Olav’s, as well as a temporal validation cohort from OUS, were used to evaluate the Oslo model (Supplementary Table S6–8). When combining all three cohorts, we observed an AUC of 0.75, *R*^2^ of 0.20, and a scaled Brier score of 0.15, which are comparable to the values from the internal predictive performance evaluation of the Oslo model. According to the calibration plot in Figure 2, the Oslo model somewhat underestimated the predicted probabilities for the HSR cohort and performed inadequately in the St. Olav’s cohort. There was a tendency to overestimate the predicted probabilities when tested on the temporal OUS cohort. The performance measures are presented in Table 3.

**Table 3:** Performance evaluation of the Oslo model using external and temporal cohorts (95% CI).

Measure	HSR^1^ cohort	St. Olav’s^2^ cohort	OUS^3^ cohort	All cohorts
AUC	0.83 (0.75, 0.91)	0.54 (0.43, 0.64)	0.80 (0.74, 0.86)	0.75 (0.70, 0.80)
*R* ^2^ *	0.30 (0.13, 0.43)	-0.29 (-0.68, -0.06)	0.31 (0.15, 0.43)	0.20 (0.07, 0.31)
Brier	0.11 (0.07, 0.14)	0.21 (0.17, 0.26)	0.15 (0.13, 0.18)	0.15 (0.13, 0.17)
Brier scaled	0.22 (0.00, 0.47)	-0.17 (-0.46, 0.07)	0.20 (0.08, 0.34)	0.15 (0.05, 0.27)

1 HSR: I.R.C.C.S. Ospedale San Raffaele (2016–2019);

2 St.Olavs: St.Olavs Hosptial (2014–2024);

3 OUS: Oslo University Hospital (2023–2024).

* Adjusted *R*^2^ Nagelkerke.

### External Validation of previously published PLND models

Patients from the development OUS cohort with complete information for all the predictors included in the Briganti 2019 model (*n* = 785) were used for the external validations. The assessment of the MSKCC, Briganti 2019, and Briganti 2019 model 2 nomograms performance is presented in Table 4. The MSKCC model showed low accuracy in our cohort. This was also reflected in the Brier scores calculated for the model, where a Brier score of 0.25 is the score of a noninformative model, and 0 is a perfect score, in a cohort with a pN1 prevalence of 50%. The Brier scale, with a range from 0 to 1, is adjusted for the prevalence in the tested cohort, and a higher value is associated with a better model performance [26]. The Briganti 2019 model that also includes MRI predictors had better discrimination and overall accuracy as measured by AUC and Brier scores, compared to MSKCC. The calibration plots for MSKCC indicate that the model was underestimating the risk in patients with predicted probabilities below 15% in our cohort (Supplementary Figure S9). Overestimation was the general trend in Briganti 2019, but this was reduced when using Briganti 2019 model 2, as observed in the calibration plots in Supplementary Figure S10 and S11. Since the clinical data in our cohort did not differentiate between MRI targeted and systematic biopsies, we used total biopsy cores to calculate the % of positive cores as a surrogate when validating Briganti 2019. But we also include model 2 from the Briganti 2019 study which do not include % positive systematic cores.

**Table 4:** Performance evaluation of the validated prediction models using the OUS development cohort (95% CI).

Measure	MSKCC	Briganti 2019	Briganti 2019 model 2
AUC	0.66 (0.61, 0.70)	0.74 (0.70, 0.78)	0.74 (0.70, 0.78)
*R* ^2^ *	0.00 (-0.27, 0.11)	0.00 (-0.14, 0.12)	0.15 (0.06, 0.23)
Brier	0.18 (0.17, 0.20)	0.19 (0.17, 0.20)	0.17 (0.15, 0.18)
Brier scaled	0.05 (0.00, 0.14)	0.04 (-0.04, 0.11)	0.13 (0.06, 0.21)

OUS: Oslo University Hospital; MSKCC: Memorial Sloan Kettering Cancer Centre.

* *R*^2^ Nagelkerke.

### Recalibration and revision of the Briganti 2019 model

To further validate the Briganti 2019 model, we used recalibration and revision to update the Briganti 2019 model based on the OUS cohort. The results from recalibrations are shown in Supplementary Table S9. The overall performance of the Briganti 2019 model improved after recalibration as seen in the change in both the *R*^2^ (from 0.0 to 0.168) and the Brier score (from 0.19 to 0.17). The calibration plots clearly showed a reduction in overestimation of the predicted probabilities and overall performance after updating the Briganti 2019 model using the OUS cohort (Supplementary Figure S12).

### Comparison of the Oslo model to Briganti 2019

**Figure 3:**
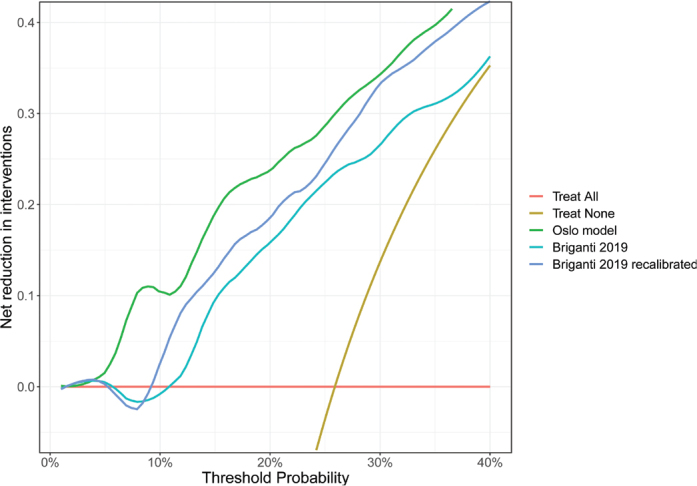
Decision curve analysis with net reduction in PLND interventions when using the Oslo model, Briganti 2019, and recalibrated Briganti 2019 model. This figure shows the development OUS cohort 2015–2022 with complete cases. PLND: pelvic lymph node dissection; OUS: Oslo University Hospital.

To quantify the difference in clinical utility between the Oslo and Briganti 2019 models we used decision curve analyses. As shown in the net reduction plot (Figure 3), the Oslo model had higher net benefit than the Briganti 2019 model at all threshold probabilities > 4%. As an example, one could use a predicted probability of 7% as a preferred risk threshold, which would result in a net reduction in interventions of 10% when using the Oslo model. The Briganti 2019 model had a net reduction of 0% at this risk threshold in the OUS cohort. Recalibrating the Briganti 2019 model using the OUS cohort gain a small shift in the net reduction but still 0% at the 7% cut-off. Supplementary Table S10 shows the classification statistics for selected risk thresholds that are covering the cut-off points used by current LNI prediction models. Plot showing the net benefit and ROC curves are provided in Supplementary Figure S13 and S14.

## Discussion and conclusion

The Oslo model demonstrated enhanced overall predictive performance and net benefit in comparison to the Briganti 2019 model. As an example, by applying a risk threshold cut-off at 10%, 185 out of 903 patients had a predicted probability below this cut-off value and could, theoretically, omit the lymph node dissection with benefits in terms of operative time, complications, and readmissions. Among these patients, 71% were in the high-risk group, and only 5.3% of them were identified as lymph node positive. These findings suggest that the Oslo model could be used to determine which high-risk patients might not need LNI staging due to the low risk of finding positive nodes. An updated model should be able to include the high-risk group in the decision-making process, instead of a treat all approach, based on the expected prevalence of 30–40% for the presence of lymph node metastases in this group [27].

The evaluation of the Oslo model, combining all external cohorts, showed good discrimination, indicated by the AUC value of 0.75. However, the calibration plot indicates that the Oslo model was underfitted, as it had a slope greater than 1 in the HSR cohort. This could be explained by the lower prevalence of positive lymph nodes and that the cohort had a higher percentage of patients in the IR group. The evaluation of the Oslo model using the St. Olav’s cohort showed poor performance. One explanation could be the higher percentage of ISUP GG 4 and cT2 with pN1 in this cohort. The difficulty of predicting the probability for these patients is highlighted by the results from the evaluation of the Briganti 2019 model 2, using the St. Olav’s cohort, which resulted in an AUC of 0.60 and *R*^2^ of –0.28.

The results of the temporal validation were more in accordance with the internal evaluation of the Oslo model as indicated by the calibration plot slope of 0.98.

We used the OUS cohort to externally validate two LNI models that are in frequent clinical use. The Briganti 2019 prediction model performed better than the MSKCC, emphasizing the additional predictive value of including MRI related predictors such as MRI staging and maximum index lesion length. This is further emphasized by the findings by Weiber and colleagues, who reported a strong association between the *T*-stage from MRI with biochemical and metastatic recurrence [28]. Both the Briganti 2019 and our new model show that this variable has a high odds ratio in models predicting LNI.

Our validation results align with those reported by Meijer and colleagues for the MSKCC and Briganti 2019 models, as evident from comparable AUC values [29]. Both models overestimated the predictive probabilities based on calibration plots. One reason for this could be related to the variation in the prevalence of LN positive in the cohorts that ranged from 10% in the cohorts used for model developments to around 25% in both the Dutch cohort and our cohort. This variation affects the performance of the models as both positive and negative predictive values are directly linked to the prevalence of positive LNI staging. This is highlighted by the results from the recalibration of the Briganti 2019 using the OUS cohort. The updating of the model resulted in better predictive accuracy, as seen by the changes in *R*^2^ and Brier score. Considering the need to continually update clinical prediction models, the implementation of a Bayesian based prediction model would make this process more convenient [30].

Our study has several limitations, one being the use of retrospective cohorts. Another is the use of small cohorts for the external and temporal validation of the developed model. A total of 13% of the patients had missing data. This was handled by using multiple imputation datasets in the development of the models. Uncertainties and biases could be introduced by imputation. But since all except one variable had less than 3% missing values, and the variable with 10% (MRI index lesion length) missing values is not one of the driving variables of the model, this should not have a major effect on the model development.

Finally, our cohort did not include data that differentiated between MRI targeted and systematic biopsies. This was addressed by using a surrogate variable for % positive cores as well as model 2 from the Briganti study when validating the Briganti 2019 model. In other external validation studies of the Briganti 2019 model up to 50% of the cases had to be excluded because of missing data indicating that the model includes biopsy variables that are not easily available for all clinicians [29, 31]. There are two MSKCC LNI models, one with (AUC 0.837) and one without (AUC 0.830) number of total and positive biopsy cores. The small difference in AUC values does not strengthen the use of % positive cores as a predictor. The change from performing more targeted biopsies and fewer systematic biopsies, makes it more challenging to use prediction models that includes % positive biopsy cores [32].

All variables in our full version model have been used in previously developed prediction models, as reported in the meta-analysis of 42 LNI models for prostate cancer by Wang et al. [1]. The inclusion of more predictors, including PSMA PET, in combination with machine learning (ML) based methods might lead to improved models. However, the lack of model transparency and the need to fine tune hyperparameters still poses challenges for ML-based models that are more manageable in logistic regression-based models.

PSMA-PET needs to be mentioned as a promising predictor of LNI. Two predictive models including PSMA-PET as a predictor were recently published [33, 34]. These models differ in that the Amsterdam–Brisbane–Sydney model includes PSMA as a predictor, whereas the Briganti 2023 model gives the probability of LNI in patients with a negative PSMA-PET. The AUC from the external validation of the Amsterdam–Brisbane–Sydney model was 0.78 (95% CI: 0.71–0.86) and 0.81 (95% CI: 0.76–0.86) [35]. The Briganti 2023 study reported an AUC of 0.78, but the model has not been externally evaluated. Neither study reported any metrics for the overall performances of their models. A third model has also been developed, the Muehlematter model, with a reported external validation AUC of 0.79 (95% CI: 0.75–0.85) [35, 36]. With the introduction of novel predictors there are new challenges that arise. Two of the main challenges regarding PSMA-PET are who should be offered a PSMA-PET scan, and how should one treat patients with miN0 and high predicted likelihood of LNI [34]. Some of these issues are addressed in the editorial that accompanied the articles by Vis et al. and Gandaglia et al. [37]. The importance of LNI prediction models is underscored by the recent updates from the 2024 EAU congress in Paris. The new guidelines no longer recommend using nomograms without MRI variables. Instead, PSMA-PET is strongly advised for patients with localized high-risk or locally advanced disease, but no clear guidance is given on how to handle miN0 patients with high predicted likelihood of LNI [38].

A robust, validated, and regularly updated LNI model can effectively guide the decision to offer PSMA-PET and help avoid unnecessary lymphadenectomies, ultimately improving quality of life and reducing health expenses.

The Oslo model is well-suited for predicting LNI and could help minimizing overtreatment in high-risk prostate cancer patients with low risk of lymph node metastasis.

## Supplementary Material



## Data Availability

Data from the Research Registry of Prostate Cancer at the Oslo University Hospital (OUS) can be made available by applying to the steering board of the research registry.
