# Supplementary material for: Development and evaluation of a lymph node invasion risk prediction model in intermediate- and high-risk prostate cancer patients
Source: Acta Oncol. 2025 Oct 22;64:43970. doi: 10.2340/1651-226X.2025.43970 (PMC12560406; doi:10.2340/1651-226X.2025.43970)

Supplementary material has been published as submitted. It has not been copyedited, or typeset by Acta Oncologica

Supplementary Methods

The TRIPOD+AI guidelines were followed to the best of our abilities and the relevant pages in the manuscript referencing to the different items on the TRIPOD+AI checklist is provided in the attachment bellow. The Oslo model and code is available at *https://hakonrlab.github.io*

TRIPOD+AI

Version: 11-January-2024

Section/Topic	Item	Development / evaluation[1]	Checklist item	Reported on page
**TITLE**				
Title	1	D;E	Identify the study as developing or evaluating the performance of a multivariable prediction model, the target population, and the outcome to be predicted	1
**ABSTRACT**				
Abstract	2	D;E	See TRIPOD+AI for Abstracts checklist	
**INTRODUCTION**				
Background	3a	D;E	Explain the healthcare context (including whether diagnostic or prognostic) and rationale for developing or evaluating the prediction model, including references to existing models	4
	3b	D;E	Describe the target population and the intended purpose of the prediction model in the context of the care pathway, including its intended users (e.g., healthcare professionals, patients, public)	4
	3c	D;E	Describe any known health inequalities between sociodemographic groups	
Objectives	4	D;E	Specify the study objectives, including whether the study describes the development or validation of a prediction model (or both)	4
**METHODS**				
Data	5a	D;E	Describe the sources of data separately for the development and evaluation datasets (e.g., randomised trial, cohort, routine care or registry data), the rationale for using these data, and representativeness of the data	5
	5b	D;E	Specify the dates of the collected participant data, including start and end of participant accrual; and, if applicable, end of follow-up	5
Participants	6a	D;E	Specify key elements of the study setting (e.g., primary care, secondary care, general population) including the number and location of centres	5
	6b	D;E	Describe the eligibility criteria for study participants	5
	6c	D;E	Give details of any treatments received, and how they were handled during model development or evaluation, if relevant	5
Data preparation	7	D;E	Describe any data pre-processing and quality checking, including whether this was similar across relevant sociodemographic groups	
Outcome	8a	D;E	Clearly define the outcome that is being predicted and the time horizon, including how and when assessed, the rationale for choosing this outcome, and whether the method of outcome assessment is consistent across sociodemographic groups	5, 6
	8b	D;E	If outcome assessment requires subjective interpretation, describe the qualifications and demographic characteristics of the outcome assessors	
	8c	D;E	Report any actions to blind assessment of the outcome to be predicted	
Predictors	9a	D	Describe the choice of initial predictors (e.g., literature, previous models, all available predictors) and any pre-selection of predictors before model building	7
	9b	D;E	Clearly define all predictors, including how and when they were measured (and any actions to blind assessment of predictors for the outcome and other predictors)	7
	9c	D;E	If predictor measurement requires subjective interpretation, describe the qualifications and demographic characteristics of the predictor assessors	6
Sample size	10	D;E	Explain how the study size was arrived at (separately for development and evaluation), and justify that the study size was sufficient to answer the research question. Include details of any sample size calculation	7
Missing data	11	D;E	Describe how missing data were handled. Provide reasons for omitting any data	7, 8
Analytical methods	12a	D	Describe how the data were used (e.g., for development and evaluation of model performance) in the analysis, including whether the data were partitioned, considering any sample size requirements	7, 8
	12b	D	Depending on the type of model, describe how predictors were handled in the analyses (functional form, rescaling, transformation, or any standardisation).	7, 8
	12c	D	Specify the type of model, rationale[2], all model-building steps, including any hyperparameter tuning, and method for internal validation	7, 8
	12d	D;E	Describe if and how any heterogeneity in estimates of model parameter values and model performance was handled and quantified across clusters (e.g., hospitals, countries). See TRIPOD-Cluster for additional considerations[3]	
	12e	D;E	Specify all measures and plots used (and their rationale) to evaluate model performance (e.g., discrimination, calibration, clinical utility) and, if relevant, to compare multiple models	7, 8
	12f	E	Describe any model updating (e.g., recalibration) arising from the model evaluation, either overall or for particular sociodemographic groups or settings	8
	12g	E	For model evaluation, describe how the model predictions were calculated (e.g., formula, code, object, application programming interface)	8, 9
Class imbalance	13	D;E	If class imbalance methods were used, state why and how this was done, and any subsequent methods to recalibrate the model or the model predictions	
Fairness	14	D;E	Describe any approaches that were used to address model fairness and their rationale	8
Model output	15	D	Specify the output of the prediction model (e.g., probabilities, classification). Provide details and rationale for any classification and how the thresholds were identified	

[1] D=items relevant only to the development of a prediction model; E=items relating solely to the evaluation of a prediction model; D;E=items applicable to both the development and evaluation of a prediction model

[2] Separately for all model building approaches.

[3] TRIPOD-Cluster is a checklist of reporting recommendations for studies developing or validating models that explicitly account for clustering or explore heterogeneity in model performance (eg, at different hospitals or centres). Debray et al, BMJ 2023; 380: e071018 [DOI: 10.1136/bmj-2022-071018]

Training versus evaluation	16	D;E	Identify any differences between the development and evaluation data in healthcare setting, eligibility criteria, outcome, and predictors	14
Ethical approval	17	D;E	Name the institutional research board or ethics committee that approved the study and describe the participant-informed consent or the ethics committee waiver of informed consent	5
**OPEN SCIENCE**				
Funding	18a	D;E	Give the source of funding and the role of the funders for the present study	18
Conflicts of interest	18b	D;E	Declare any conflicts of interest and financial disclosures for all authors	
Protocol	18c	D;E	Indicate where the study protocol can be accessed or state that a protocol was not prepared	
Registration	18d	D;E	Provide registration information for the study, including register name and registration number, or state that the study was not registered	5
Data sharing	18e	D;E	Provide details of the availability of the study data	
Code sharing	18f	D;E	Provide details of the availability of the analytical code[4]	26
**PATIENT & PUBLIC INVOLVEMENT**				
Patient & Public Involvement	19	D;E	Provide details of any patient and public involvement during the design, conduct, reporting, interpretation, or dissemination of the study or state no involvement.	
**RESULTS**				
Participants	20a	D;E	Describe the flow of participants through the study, including the number of participants with and without the outcome and, if applicable, a summary of the follow-up time. A diagram may be helpful.	Supp Fig1
	20b	D;E	Report the characteristics overall and, where applicable, for each data source or setting, including the key dates, key predictors (including demographics), treatments received, sample size, number of outcome events, follow-up time, and amount of missing data. A table may be helpful. Report any differences across key demographic groups.	Tab 1<br>Supp T1
	20c	E	For model evaluation, show a comparison with the development data of the distribution of important predictors (demographics, predictors, and outcome).	Tab 1
Model development	21	D;E	Specify the number of participants and outcome events in each analysis (e.g., for model development, hyperparameter tuning, model evaluation)	10
Model specification	22	D	Provide details of the full prediction model (e.g., formula, code, object, application programming interface) to allow predictions in new individuals and to enable third-party evaluation and implementation, including any restrictions to access or re-use (e.g., freely available, proprietary)[5]	26
Model performance	23a	D;E	Report model performance estimates with confidence intervals, including for any key subgroups (e.g., sociodemographic). Consider plots to aid presentation.	Tab 2
	23b	D;E	If examined, report results of any heterogeneity in model performance across clusters. See TRIPOD Cluster for additional details[3].	Fig 2
Model updating	24	E	Report the results from any model updating, including the updated model and subsequent performance	
**DISCUSSION**				
Interpretation	25	D;E	Give an overall interpretation of the main results, including issues of fairness in the context of the objectives and previous studies	14
Limitations	26	D;E	Discuss any limitations of the study (such as a non-representative sample, sample size, overfitting, missing data) and their effects on any biases, statistical uncertainty, and generalizability	16
Usability of the model in the context of current care	27a	D	Describe how poor quality or unavailable input data (e.g., predictor values) should be assessed and handled when implementing the prediction model	16, 17
	27b	D	Specify whether users will be required to interact in the handling of the input data or use of the model, and what level of expertise is required of users	26
	27c	D;E	Discuss any next steps for future research, with a specific view to applicability and generalizability of the model	16, 17

From: Collins GS, Moons KGM, Dhiman P, et al. *BMJ* 2024;385:e078378. doi:10.1136/bmj-2023-078378

[4] This relates to the analysis code, for example, any data cleaning, feature engineering, model building, evaluation.
[5] This relates to the code to implement the model to get estimates of risk for a new individual.

Supplementary Tables

Supplementary Table S1: EAU risk group stratification between pN0 and pN1 patients of the OUS development cohort

	Lymph node status		
**Variable**	pN0 (n = 663)	pN1 (n = 240)	p-value[1]
**EAU risk groups**			<0.001
**Low-risk**	4/636 (0.6%)	5/223 (2.2%)	
**Intermediate-risk**	149/636 (23%)	36/223 (16%)	
**High-risk**	395/636 (62%)	124/223 (56%)	
**Locally advanced**	88/636 (14%)	58/223 (26%)	
**Missing**	27	17	

1 Fisher´s exact test

Supplementary Table S2: Sample size calculations to estimate the minimum number of samples and events required.

	**Sample size**	**Shrinkage**	**Parameter**	**R^2 CS[1]**	**R^2 Max[2]**	**R^2 Nag[3]**	**EEP[4]**
**Criteria 1**	745	0.90	20	0.21	0.68	0.31	9.69
**Criteria 2**	511	0.86	20	0.21	0.68	0.31	6.64
**Criteria 3**	296	0.90	20	0.21	0.68	0.31	3.85
**Final**	745	0.90	20	0.21	0.68	0.31	9.69

[1] R^2 CS = R^2 Cox and Snell
[2] R^2 Max = R^2 maximum
[3] R^2 Nag = R^2 Nagelkerke
[4] EPP = events per predictor

Criteria 1: small overfitting defined by an expected shrinkage of predictor effects (default: by 10% or less).
Criteria 2: small absolute difference (default: 0.05) in the model's apparent and adjusted Nagelkerke's R-squared value.
Criteria 3: precise estimation (default: within +/- 0.05) of the average outcome risk in the population for a key timepoint of interest for prediction.

Supplementary Table S3: Baseline clinical characteristics of the OUS development cohort

	**Complete**	**Missing**	
**Variable**	n = 785[1]	n = 118[1]	p-value[2]
**Age at surgery**	68.00 (63.00, 72.00)[1]	67.00 (62.00, 71.00)	0.2
**Pre-surgery PSA**	9.10 (6.70, 14.70)	10.00 (7.23, 17.98)	0.075
**ISUP grade group**			0.007
6	3 / 785 (0.4%)	3 / 118 (2.5%)	
7a	52 / 785 (6.6%)	10 / 118 (8.5%)	
7b	213 / 785 (27%)	21 / 118 (18%)	
8	288 / 785 (37%)	39 / 118 (33%)	
9	213 / 785 (27%)	39 / 118 (33%)	
10	16 / 785 (2.0%)	6 / 118 (5.1%)	
**cT-stage (DRE)**			0.3
T1a	1 / 785 (0.1%)	0 / 108 (0%)	
T1c	308 / 785 (39%)	37 / 108 (34%)	
T2	18 / 785 (2.3%)	5 / 108 (4.6%)	
T2a	190 / 785 (24%)	20 / 108 (19%)	
T2b	90 / 785 (11%)	15 / 108 (14%)	
T2c	46 / 785 (5.9%)	6 / 108 (5.6%)	
T3a	117 / 785 (15%)	17 / 108 (16%)	
T3b	8 / 785 (1.0%)	4 / 108 (3.7%)	
TX	7 / 785 (0.9%)	4 / 108 (3.7%)	
(Missing)	0	10	
**cT-stage (MRI)**			<0.001
T1c	3 / 785 (0.4%)	13 / 118 (11%)	
T2	356 / 785 (45%)	36 / 118 (31%)	
T3a	315 / 785 (40%)	35 / 118 (30%)	

T3b	108 / 785 (14%)	30 / 118 (25%)	
T4	3 / 785 (0.4%)	4 / 118 (3.4%)	
**Max lesion length (MRI)**	19.00 (13.00, 26.00)	17.00 (11.00, 23.50)	0.4
(Missing)	0	91	
**pN**			0.2
pN0	582 / 785 (74%)	81 / 118 (69%)	
pN1	203 / 785 (26%)	37 / 118 (31%)	
**No. tot biopsy cores**	10 (7, 11)	10 (7, 10)	0.2
(Missing)	0	8	
**No. positive biopsy cores**	5 (3, 7)	5 (3, 7)	0.3
(Missing)	0	18	
**Prostate volume**	38.00 (30.00, 50.00)	35.00 (30.00, 46.00)	0.2
(Missing)	0	7	
**BMI**	26.40 (24.38, 29.20)	27.40 (25.13, 29.07)	0.11
(Missing)	0	4	
**Missing data**	0 / 0 (0%)	118 / 118 (100%)	
(Missing)	785	0	

[1]Median (IQR); n/N (%)
[2]Wilcoxon rank sum test; Fisher's exact test; Pearson's Chi-squared test

Supplementary Table S4: Table of β coefficients in the final Oslo model

	**Mean *β***	**S.E.**
**Intercept**	-2.41	4.46
**PSA**	0.09	0.70
**PSA’**	-7.37	8.50
**PSA”**	15.90	17.10
**Prostate volume**	-0.02	0.17
**Prostate volume’**	-1.03	2.03
**Prostate volume”**	2.79	4.29
**ISUP grade group 3**	0.27	0.37
**ISUP grade group 4**	0.31	0.37
**ISUP grade group 5**	1.11	0.36
**MRI cT3a**	0.60	0.23
**MRI cT3b**	1.85	0.29
**MRI index lesion length**	0.01	0.06
**MRI index lesion length’**	0.34	0.32
**MRI index lesion length”**	-0.93	0.71
**PSA × Prostate volume**	-0.00	0.03
**PSA’ × Prostate volume**	0.30	0.31
**PSA” × Prostate volume**	-0.66	0.62
**PSA × Prostate volume’**	0.22	0.31
**PSA’ × Prostate volume’**	-4.68	3.49
**PSA” × Prostate volume’**	9.70	6.87
**PSA × Prostate volume”**	-0.55	0.66
**PSA’ × Prostate volume”**	10.24	7.28
**PSA” × Prostate volume”**	-21.07	14.31

ISUP grade groups 1-2 and MRI T2 are the reference groups for these two variables.
‘ Knot-1 from the natural splines that were used for PSA, prostate volume and MRI index lesion length.
‘’ Knot-2 from the natural splines that were used for PSA, prostate volume MRI index lesion length

Supplementary Table S5: Expected log-predictive density (ELPD) estimates of the full and final models

	**Full Oslo model**		**Final Oslo model**	
	Estimates	S.E.	Estimates	S.E.
**ELPD loo***	-463.3	18.4	-457.2	17.4
**p§ loo**	4.7	3.5	26.8	2.4
**looic****	927.7	36.7	914.3	34.8
**ELPD diff**	-6.7	4.9	0.0	0.0

*loo = leave-one-out-cross-validation
§ p = effective number of parameter
** looic = the LOO information criterion

Supplementary Table S6: Baseline clinical characteristics of the HSR cohort

	Lymph node status		
**Variable**	pN0 (n = 159)	pN1 (n = 30)	p-value[2]
**Pre-surgery PSA (ng/mL)**	7.0 (4.8, 10.0)[1]	10.1 (6.5, 17.0)	<0.001
**ISUP grade group**			<0.001
**1**	14 / 159 (7.4%)	0 / 30 (0%)	
**2**	56 / 159 (35%)	1 / 30 (3.3%)	
**3**	55 / 159 (30%)	7 / 30 (23%)	
**4**	34 / 159 (17%)	7 / 30 (23%)	
**5**	30 / 159 (16%)	15 / 30 (50%)	
**cT-stage (MRI)**			<0.001
**≤T2**	135 / 159 (85%)	15 / 30 (50%)	
**T3a**	19 / 159 (12%)	9 / 30 (30%)	
**≥T3b**	5 / 159 (3%)	6 / 30 (20%)	
**Max lesion length (MRI)(mm)**	12 (8, 15)	18 (15, 20)	<0.001
**Prostate volume (cc)**	42 (35, 55)	42 (35, 55)	0.9

[1]Median (IQR); n / N (%)
[2]Wilcoxon rank sum test; Pearson's Chi-squared test; Fisher's exact test

Supplementary Table S7: Baseline clinical characteristics of the St.Olavs cohort

	Lymph node status		
**Variable**	pN0 (n = 118)	pN1 (n = 39)	p-value[2]
**Pre-surgery PSA (ng/mL)**	9.2 (6.7, 17.0)[1]	9.8 (4.8, 24.7)	0.80
**ISUP grade group**			0.41
**1**	1 / 118 (0.8%)[1]	1 / 39 (2.6%)	
**2**	24 / 118 (20.3%)	4 / 39 (10.2%)	
**3**	44 / 118(37.3%)	12 / 39 (30.8%)	
**4**	35 / 118 (29.7%)	16 / 39 (41.0%)	
**5**	14 / 118 (11.9%)	6 / 39 (15.4%)	
**cT-stage (MRI)**			0.23
**≤T2**	66 / 118 (55.9%)	23 / 39 (59%)	
**T3a**	43 / 118 (35.6%)	10 / 39 (25.6%)	
**≥T3b**	9 / 118 (7.6%)	6 / 39 (15.4%)	
**Max lesion length (MRI)(mm)**	17 (12, 24)	22 (16, 35)	0.002
**Prostate volume (cc)**	37 (29, 46)	45 (36, 52.5)	0.006
**EAU risk groups**			0.61
**Low-risk**	0 / 118 (0%)	0 / 39 (0%)	
**Intermediate-risk**	21 / 118 (18%)	5 / 39 (13%)	
**High-risk**	45 / 118(38%)	18 / 39 (46%)	
**Locally advanced**	52 / 118 (44%)	16 / 39 (41%)	

[1]Median (IQR); n / N (%)
[2]Wilcoxon rank sum test; Pearson's Chi-squared test; Fisher's exact test

Supplementary Table 8: Baseline clinical characteristics of temporal OUS cohort

	Lymph node status		
**Variable**	pN0 (n = 166)	pN1 (n = 65)	p-value[2]
**Age at surgery**	68.00 (62.00, 74.00)[1]	67.00 (63.00, 71.00)[1]	0.6
**Pre-surgery PSA (ng/mL)**	8.80 (6.25, 14.15)	11.00 (7.50, 20.00)	0.07
**ISUP grade group**			0.002
**≤2**	21 / 166 (13%)	2 / 65 (3.1%)	
**3**	50 / 166 (30%)	18 / 65 (28%)	
**4**	61 / 166 (37%)	17 / 65 (26%)	
**5**	34 / 166 (20%)	28 / 65 (43%)	
**cT -stage (DRE)**			0.2
**T1**	40 / 100 (40%)	12 / 43 (28%)	
**T2**	45 / 100 (45%)	20 / 43 (47%)	
**T3**	15 / 100 (15%)	11 / 43 (26%)	
**Missing**	6	2	
**EAU risk groups**			0.4
**Low-risk**	1 / 95 (1.1%)	0 / 41 (0%)	
**Intermediate-risk**	24 / 95 (25%)	7 / 41 (17%)	
**High-risk**	55 / 95 (58%)	23 / 41 (56%)	
**Locally advanced**	15 / 95 (16%)	11 / 41 (27%)	
**Missing**	11	4	
**PI-RADS score (MRI)**			0.002
**≤3**	11 / 104 (11%)	1 / 45 (2%)	
**4**	36 / 104 (35%)	4 / 45 (13%)	
**5**	57 / 104 (55%)	38 / 45 (85%)	
**Missing**	2	0	
**cT-stage (MRI)**			<0.001

**≤T2**	72 / 166 (43%)	3 / 65 (4.6%)	
**T3a**	76 / 166 (46%)	29 / 65 (45%)	
**≥T3b**	18 / 166 (11%)	33 / 65 (51%)	
**Max lesion length (MRI)(mm)**	18.50 (12.00, 28.00)	28.00 (21.00, 37.00)	<0.001
**Missing**	4	2	
**Prostate volume (cc)**	38.26 (32.08, 48.43)	42.00 (35.00, 52.00)	0.13
**No. of removed lymph nodes**	9.00 (7.00, 13.00)	12.00 (8.00, 15.00)	0.13
**No. of positive lymph nodes**	0 (0, 0)	2 (1, 3)	<0.001

[1]Median (IQR); n / N (%)
[2]Wilcoxon rank sum test; Pearson's Chi-squared test; Fisher's exact test

Supplementary Table S9: The predicted performance of the Briganti 2019 model after recalibration using the OUS development cohort (95% CI)

** R2 Nagelkerke*

Measure	Recalibration-in-the-large (only intercept adjusted)	Recalibration (intercept and coefficients adjusted)
**AUC**	0.74 (0.67, 0.78)	0.74 (0.70, 0.78)
**R2***	0.16 (0.10, 0.23	0.17 (0.09, 0.23)
**Brier**	0.16 (0.15, 0.18)	0.17 (0.15, 18)
**Brier scaled**	0.15 (0.08, 0.22)	0.16 (0.07, 0.22)

Supplementary Table S10: Classification statistics of the Oslo model at different selected risk thresholds (95% CI)[1]

Risk threshold	Sensitivity	Specificity	PPV*	NPV§	Overall accuracy
**< 5%**	1.00 (0.97, 1.00)	0.05 (0.03, 0.07)	0.27 (0.24, 0.30)	0.97 (0.83, 1.00)	0.30 (0.26, 33)
**< 7%**	0.99 (0.97, 0.10)	0.15 (0.12,0.18)	0.29 (0.26, 0.32)	0.98 (0.92, 1.00	0.37 (0.33, 0.40)
**<10%**	0.93 (0.89, 0.96)	0.33 (0.29, 0.37)	0.33 (0.29, 0.37)	0.93 (0.89, 0.96)	0.49 (0.45, 0.52)

* PPV = Positive predictive value
§ NPV = Negative predictive value

[1] Positive and negative predictive values are directly related to prevalence.

Supplementary figures

Supplementary Figure S1: Patient selection flowchart for the development cohort (OUS 2015-2022).

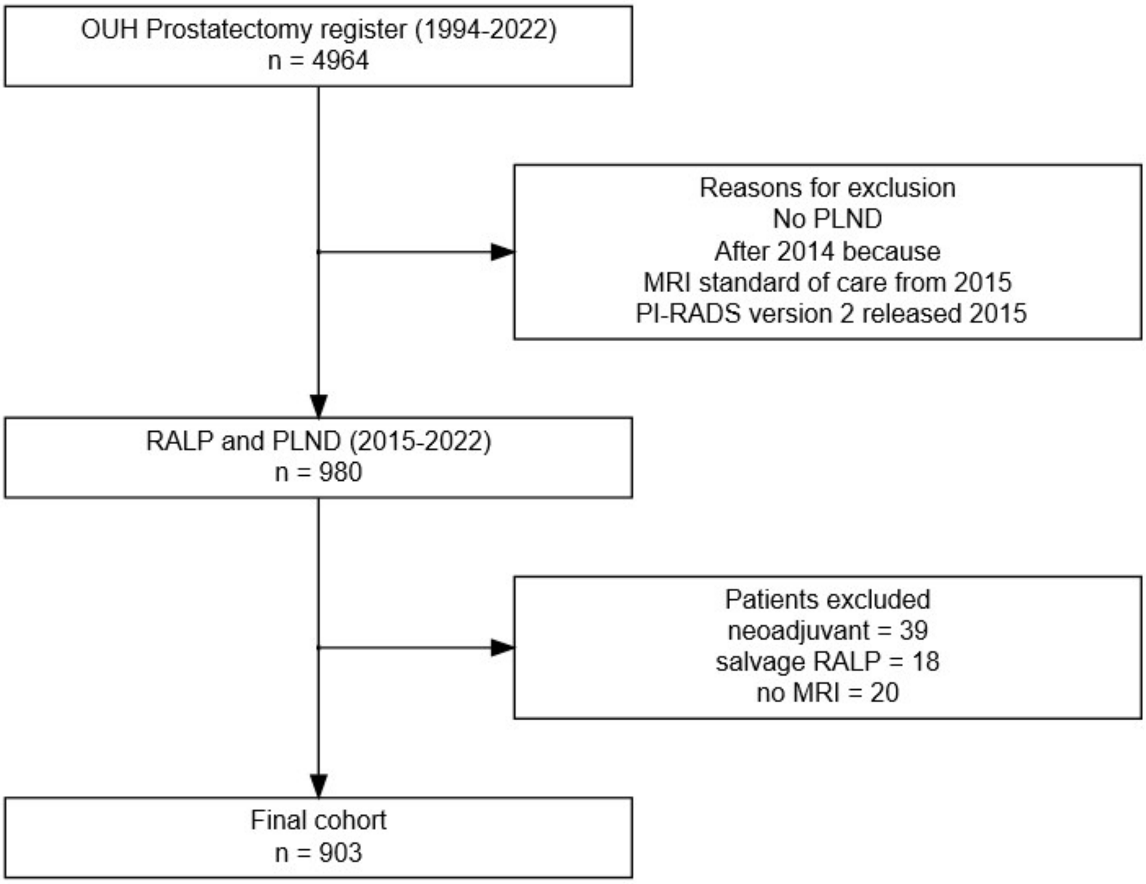

Supplementary Figure S2: Distribution of missing data for all variables in the Full model.

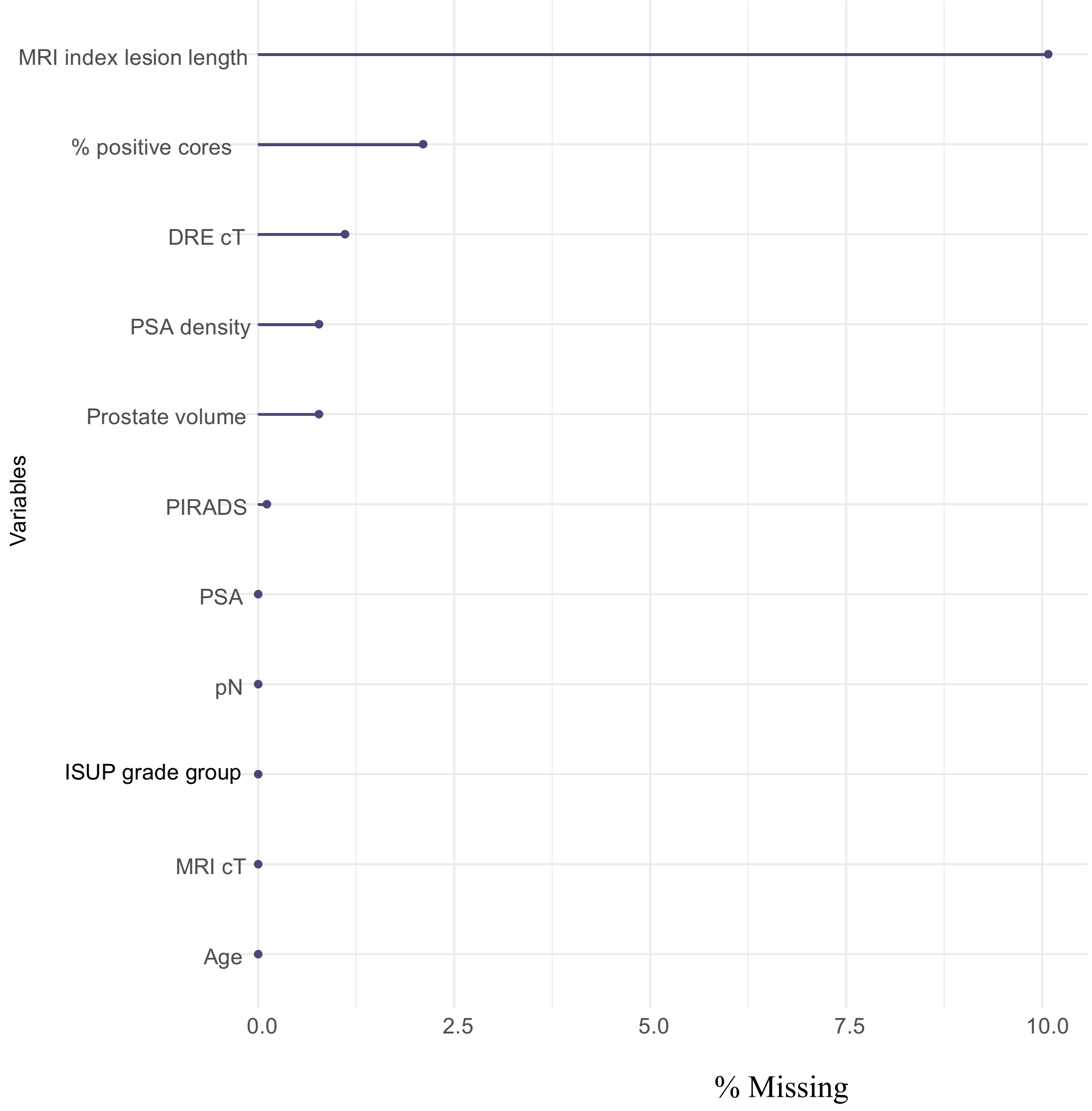

Supplementary Figure S3: Posterior distribution of coefficients in the full Oslo model. The x-axis represents the coefficient values and density is represented on the y-axis. All 10 imputed datasets are shown, and the two vertical lines represents the 95% highest posterior density interval (HPDI). The ‘and ‘’ indicates the knots from the natural splines that were used for PSA and prostate volume. PrecPosCores is the % positive cores variable.

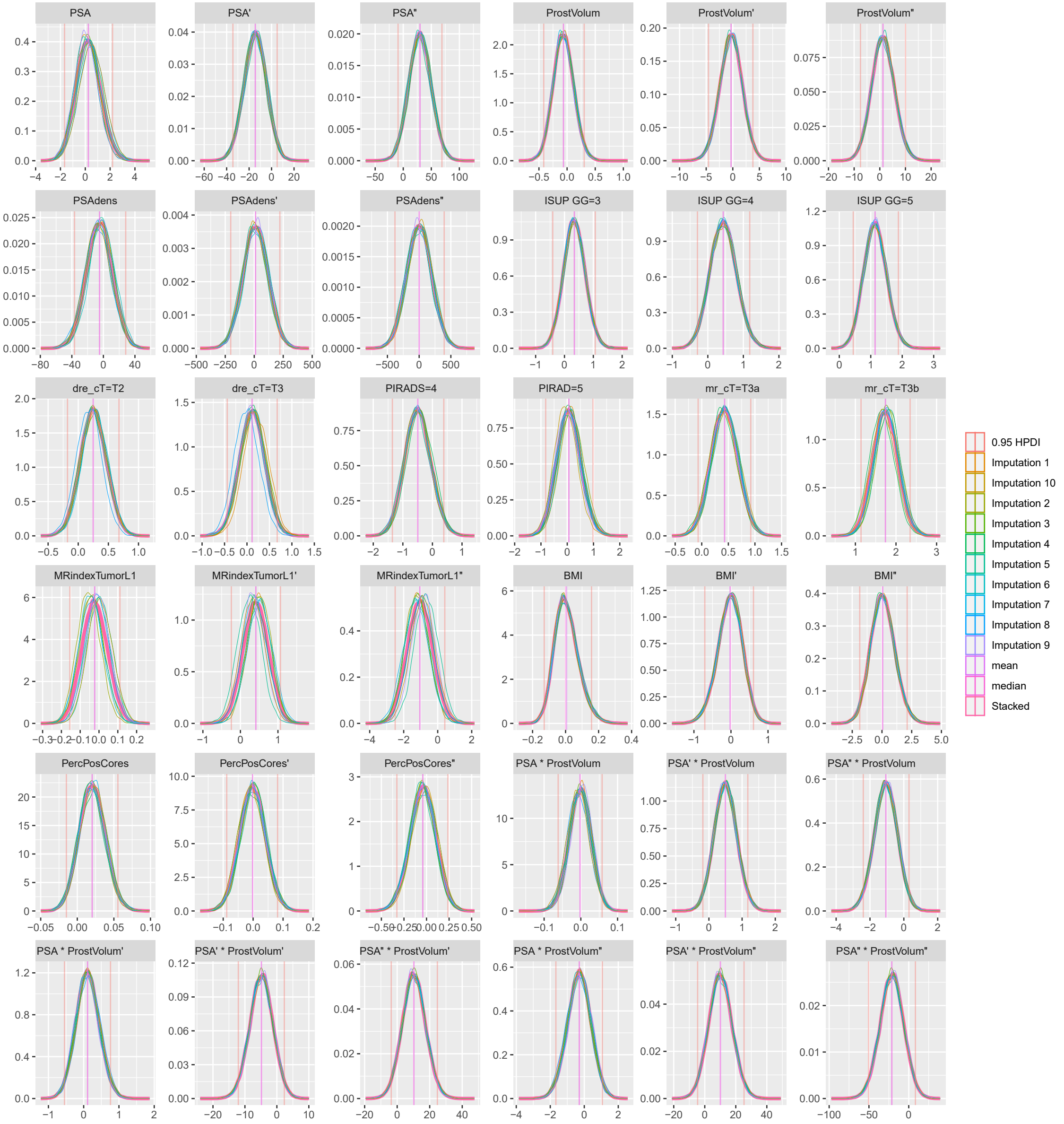

Supplementary Figure S4: Relative explained variation of the predictors in the full Oslo model using an ANOVA test.

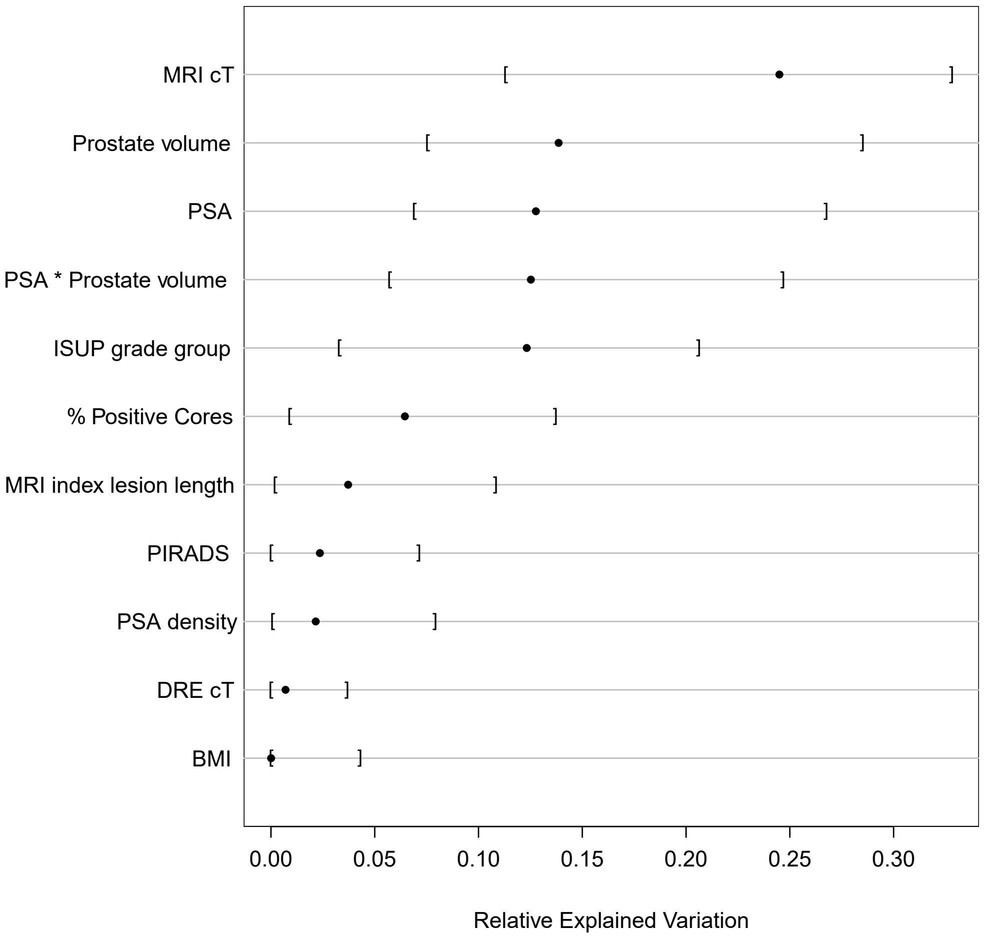

Supplementary Figure S5: Variable selection plot of the results from variable selection method used by the cv-varsel function in the projpred R package using the forward method without cross-validation.

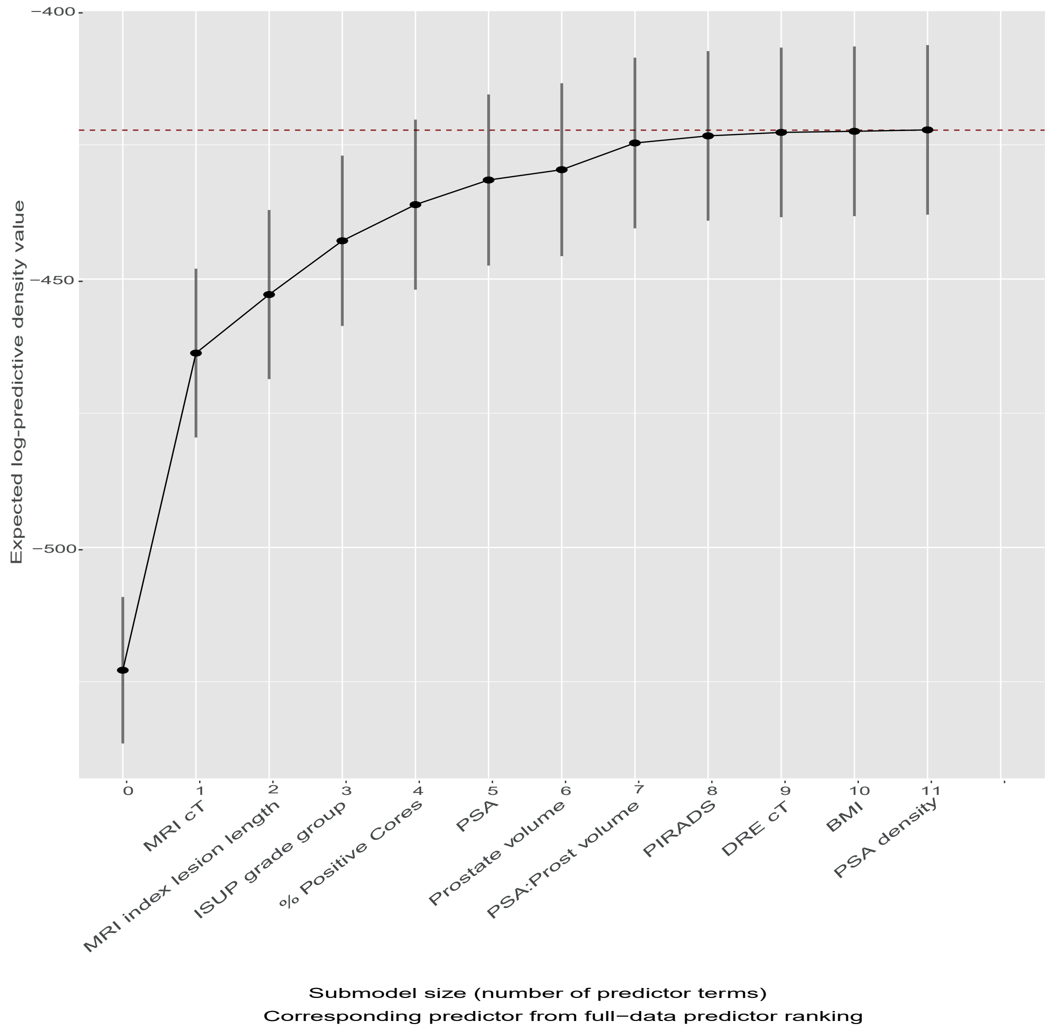

Supplementary Figure S6: Variable selection plot of the results from the cross-validation based variable selection method used by the cv-varsel function in the projpred R package.

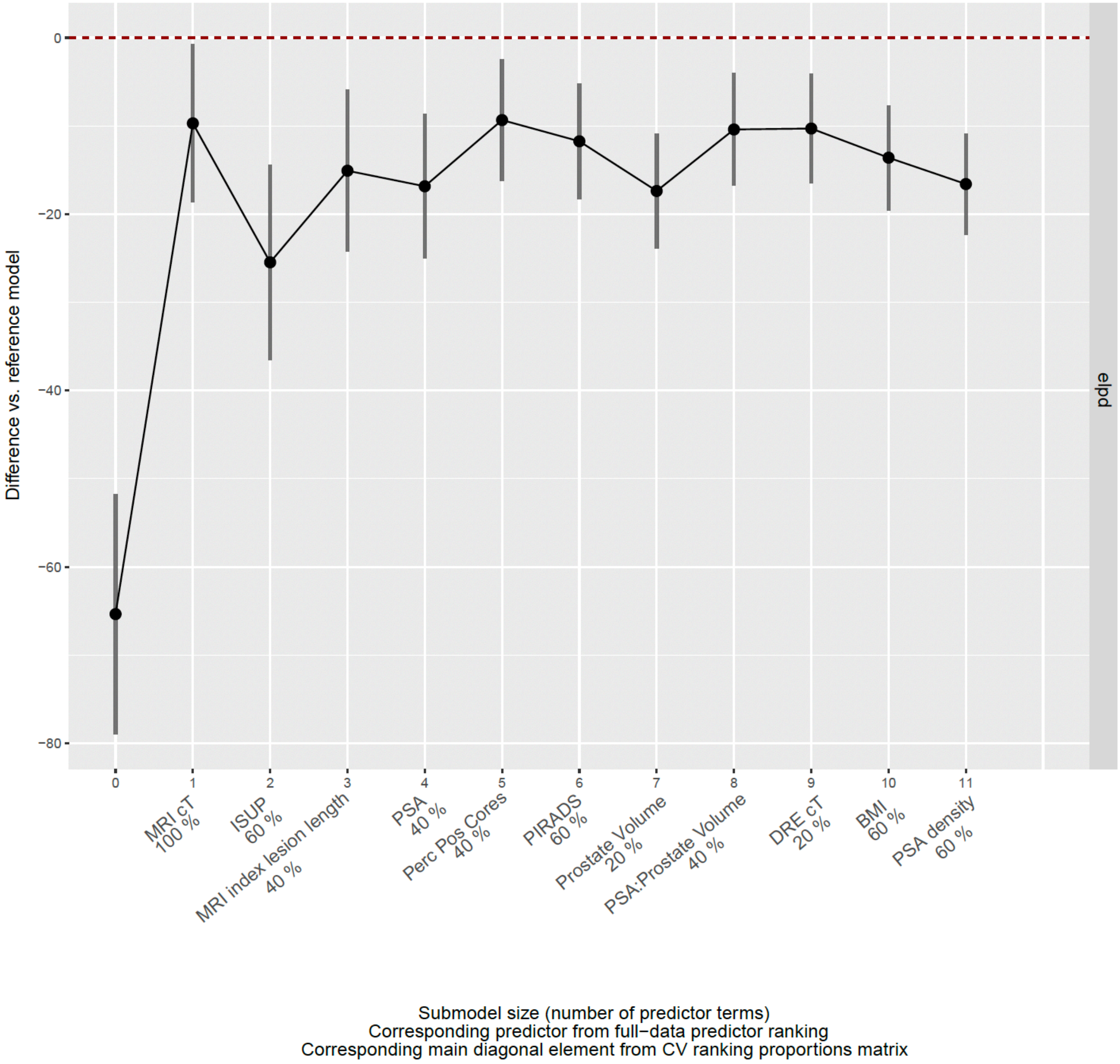

Supplementary Figure S7: Posterior distribution of coefficients in the final Oslo model.
The x-axis represents the coefficient values and density is represented on the y-axis. All 10 imputed datasets are shown and the two vertical lines represents the 95% highest posterior density interval (HPDI). The ‘ and ‘’ indicates the knots from the natural splines that were used for PSA and prostate volume.

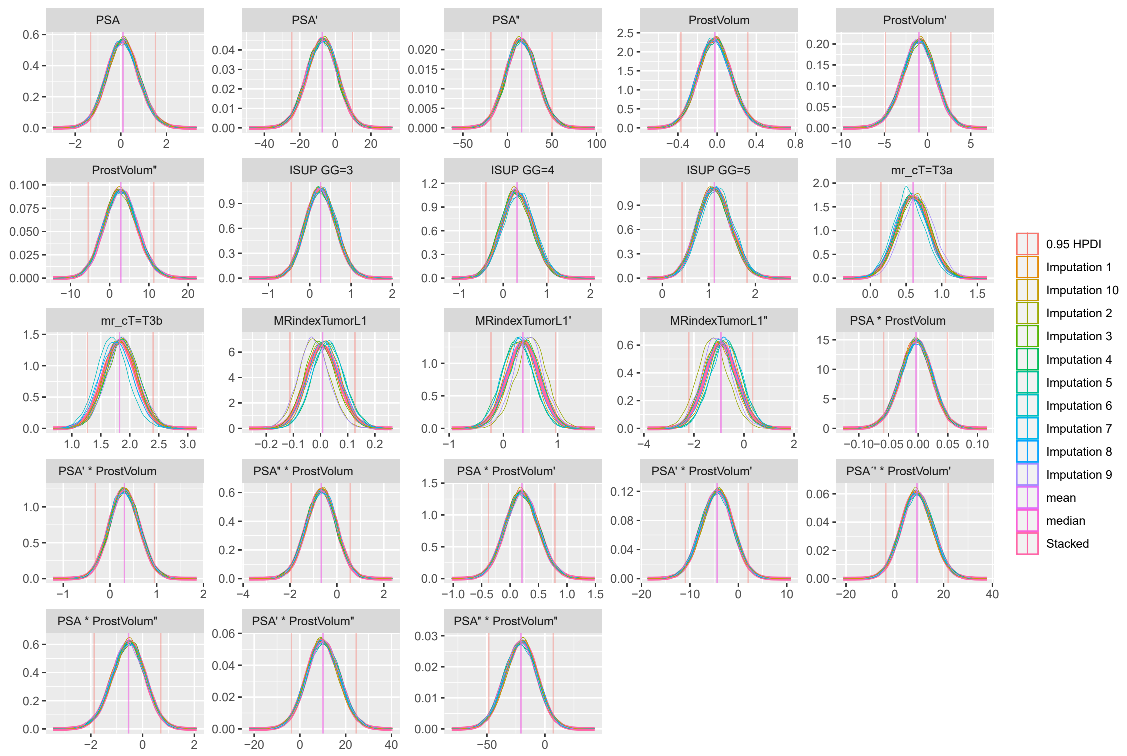

Supplementary Figure S8: Relative explained variation of predictors regarding the predicted response in the final Oslo model using an ANOVA test

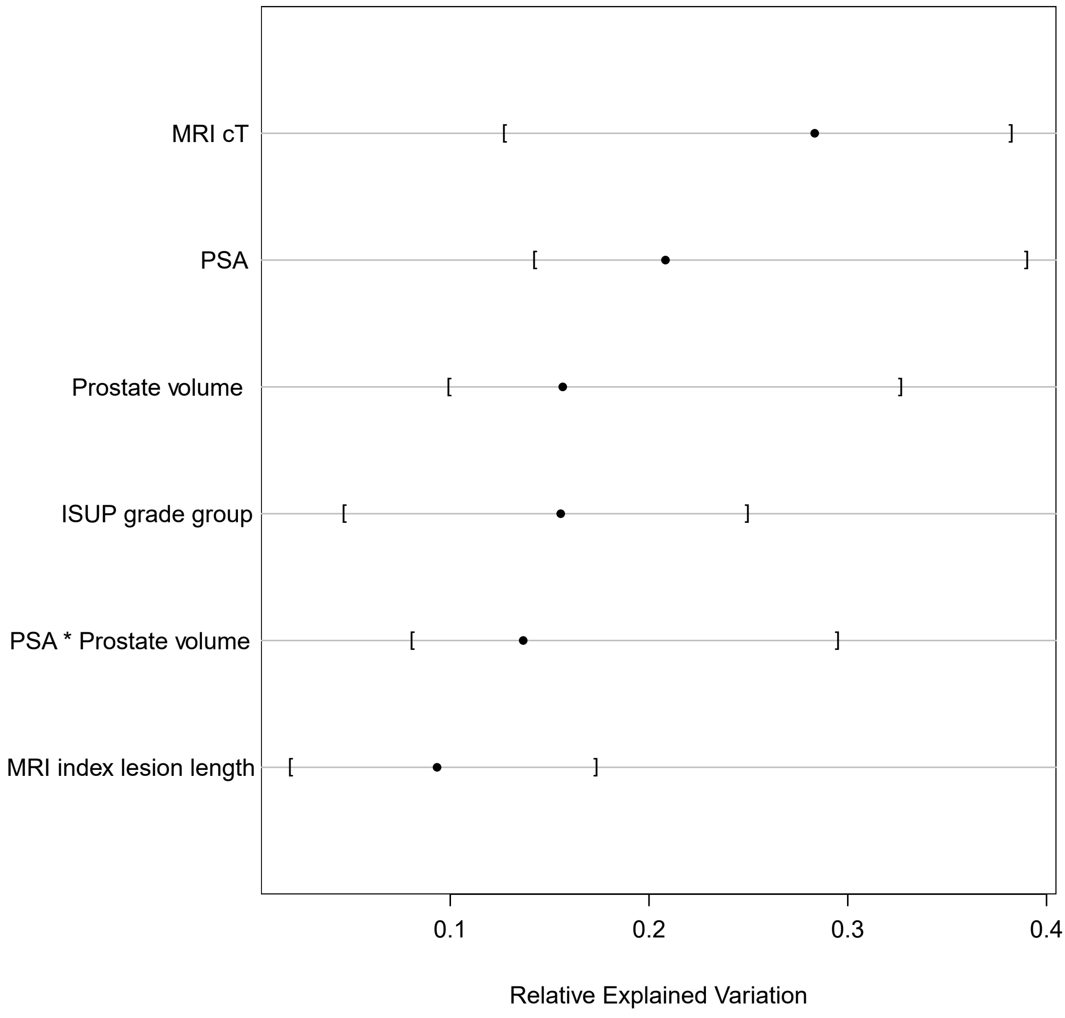

Supplementary Figure S9: MSKCC calibration plot based on the complete cases from the OUS cohort. The dashed line represents the logistic approximation, and the red line is the flexible calibration curve based on locally estimated scatterplot smoothing. Grey shaded area represents the 95% confidence interval. The diagonal black line represents an ideal calibration curve. Histograms show the distribution of predicted probabilities of pN0 and pN1 patients.

MSKCC model

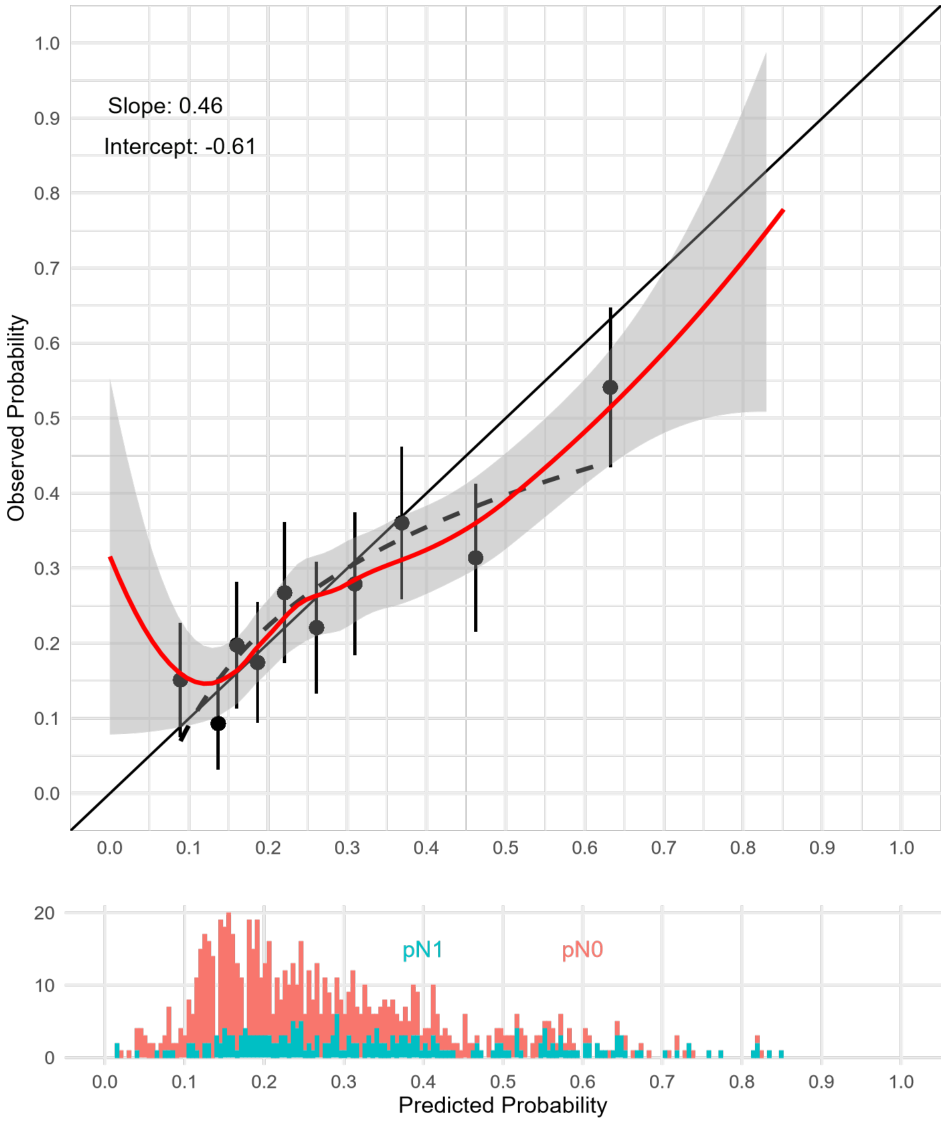

Supplementary Figure S10: Briganti 2019 calibration plot based on the complete cases from the OUS cohort. The dashed line represents the linear approximation, and the red line is the flexible calibration curve based on loess. Grey shaded area represents the 95% confidence interval. The diagonal black line represents an ideal calibration curve. Histograms show the distribution of predicted probabilities of pN0 and pN1 patients.

Briganti 2019 model

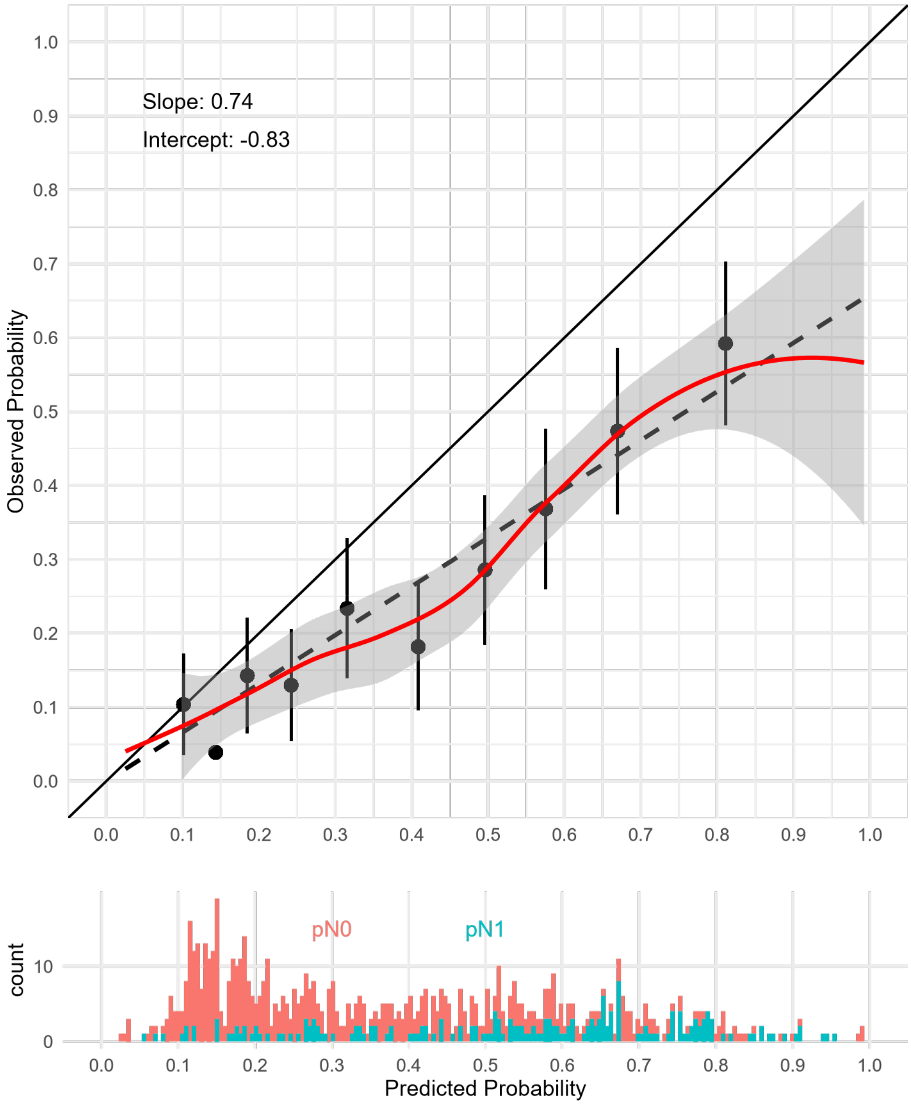

Supplementary Figure S11: Briganti 2019 model 2 calibration plot based on the complete cases from the OUS cohort. The dashed line represents the linear approximation, and the red line is the flexible calibration curve based on loess. Grey shaded area represents the 95% confidence interval. The diagonal black line represents an ideal calibration curve. Histograms show the distribution of predicted probabilities of pN0 and pN1 patients.

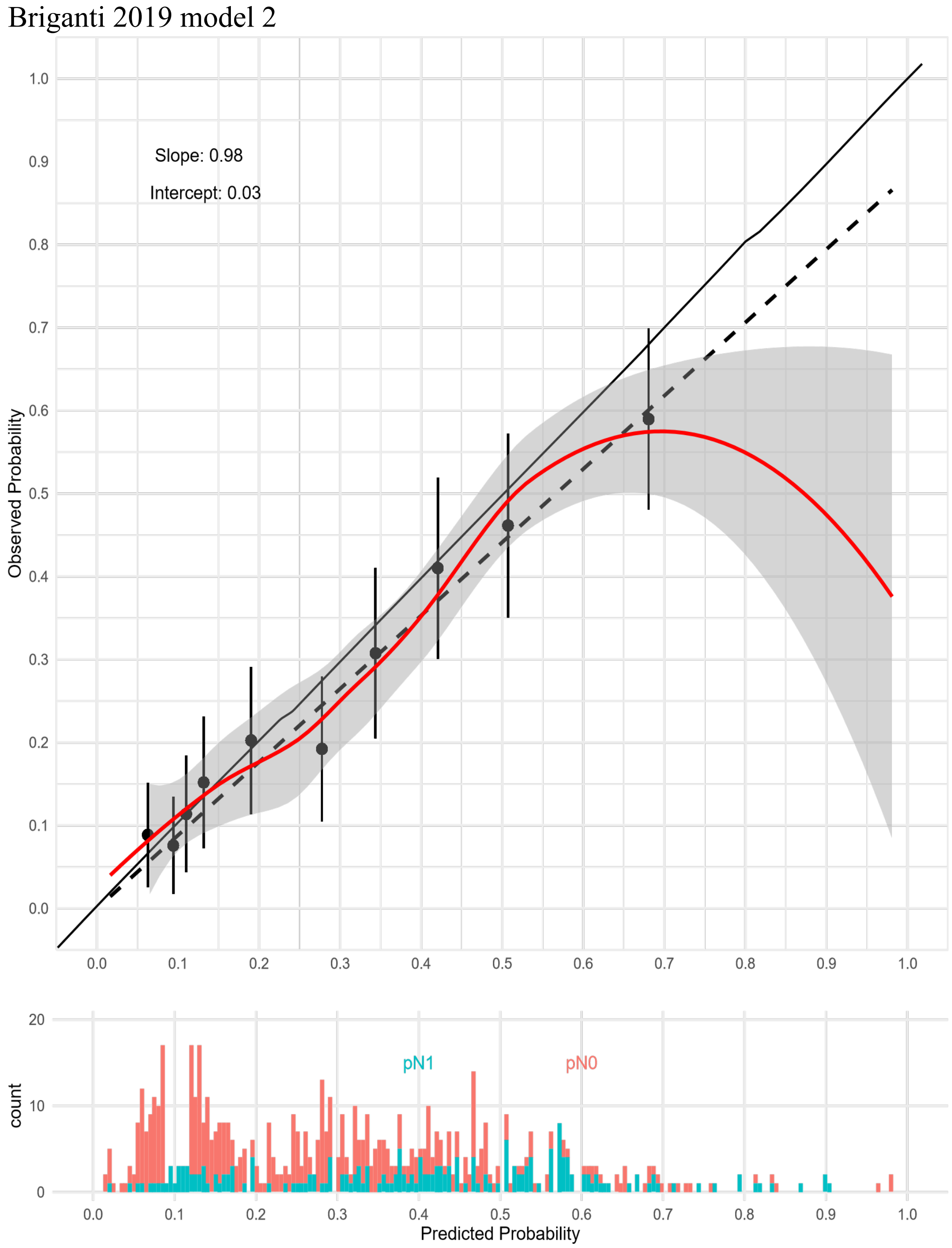

Supplementary Figure S12: Calibration plot after recalibration of Briganti 2019 model in the OUS complete cases cohort. The dashed line represents the linear approximation, and the red line is the flexible calibration curve based on loess. Grey shaded area represents the 95% confidence interval. The diagonal black line represents an ideal calibration curve. Histograms show the distribution of predicted probabilities of pN0 and pN1 patients.

Briganti 2019 recalibrated model

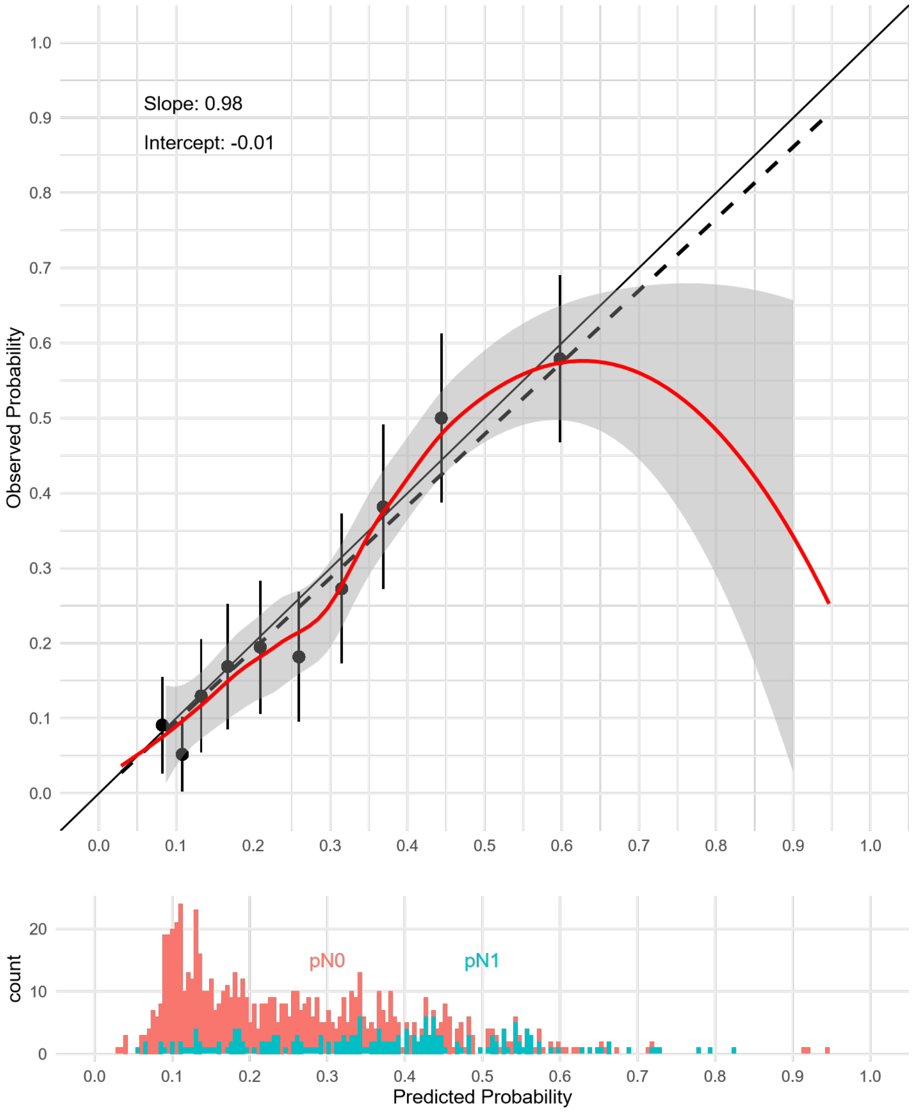

Supplementary Figure S13: Decision curves analysis with net benefit in the Oslo and Briganti 2019 models using the complete cases from the OUS cohort.

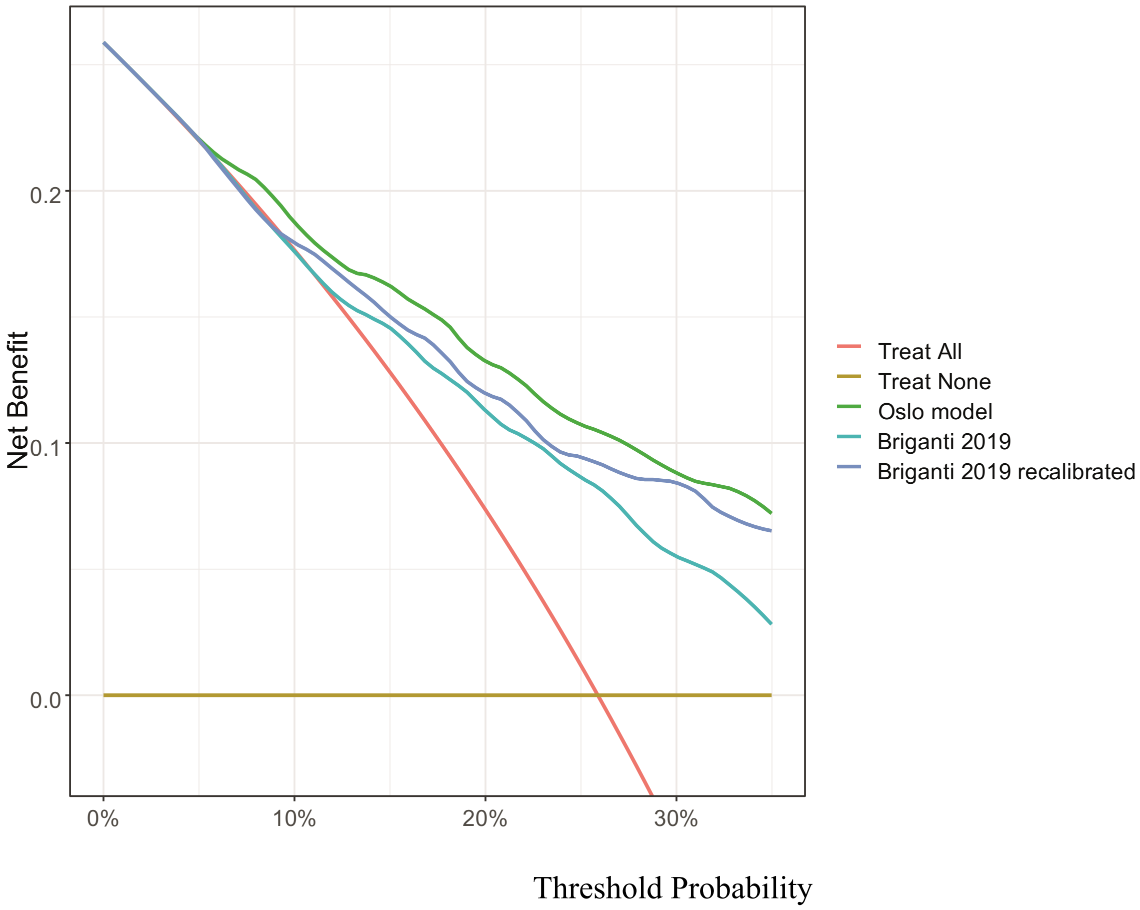

Supplementary Figure S14: ROC curves of the Oslo and Briganti 2019 models using the complete cases OUS cohort.

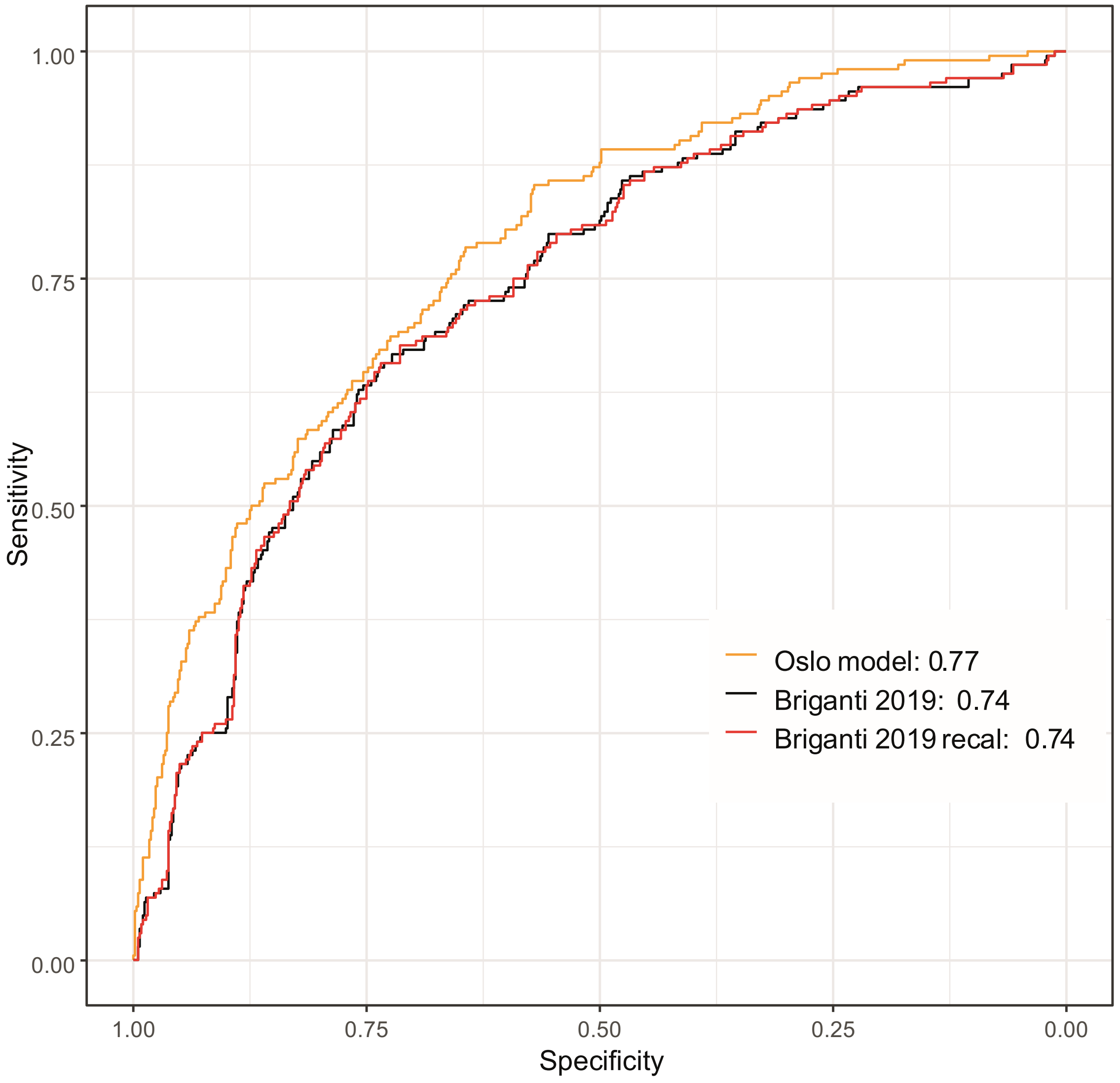

Supplement: Supplementary file 1 [file AO-64-43970-s1.pdf]
